# Association between hepatic steatosis and fibrosis with measures of insulin sensitivity in patients with severe obesity and type 2 diabetes - a cross-sectional study

**DOI:** 10.1186/s12876-022-02550-0

**Published:** 2022-11-07

**Authors:** Kathrine Aglen Seeberg, Dag Hofsø, Heidi Borgeraas, John Olav Grimnes, Farhat Fatima, Lars Thomas Seeberg, Nils Petter Kvan, Marius Svanevik, Jens Kristoffer Hertel, Jøran Hjelmesæth

**Affiliations:** 1 Morbid Obesity Centre, Vestfold Hospital Trust, Tønsberg, Norway; 2 Department of Medicine, Vestfold Hospital Trust, Tønsberg, Norway; 3 Institute of Clinical Medicine, University of Oslo, Oslo, Norway; 4 Department of Radiology, Vestfold Hospital Trust, Tønsberg, Norway; 5 Department of Surgery, Vestfold Hospital Trust, Tønsberg, Norway; 6 Department of Endocrinology, Morbid Obesity and Preventive Medicine, Institute of Clinical Medicine, University of Oslo, Oslo, Norway

**Keywords:** Severe obesity, Type 2 diabetes mellitus, Hepatic steatosis, Liver fibrosis, Insulin sensitivity, Liver fat fraction, ELF test

## Abstract

**Background:**

Obesity, non-alcoholic fatty liver disease (NAFLD) and insulin resistance are three pathological conditions highly correlated, but this relationship is not fully elucidated. Hence, we aimed to assess the association of hepatic steatosis and fibrosis with different measures of insulin sensitivity in patients with severe obesity and type 2 diabetes mellitus (T2DM).

**Methods:**

A cross-sectional study (Oseberg trial) including patients with T2DM referred for bariatric surgery at Vestfold Hospital Trust, Norway. Magnetic resonance imaging (MRI) and the enhanced liver fibrosis (ELF) test was used for estimation of liver fat fraction (LFF) and degree of fibrosis, respectively. Oral and intravenous glucose tolerance tests were applied for estimation of insulin sensitivity (HOMA2S, Matsuda ISI and MinMod SI).

**Results:**

A total of 100 patients (mean [SD] age 47.5 [9.7] years, 65% women, BMI 42.0 [5.3] kg/m^2^ and 98% with metabolic syndrome) were included in the analyses. The mean (SD) LFF in the total population was 19.1 (11.5), and the mean (SD) ELF score was 8.46 (0.84), a value representing moderate fibrosis. LFF was inversely associated with HOMA2S and Matsuda ISI, and both measures were significantly higher in the no or low-grade steatosis group compared with the medium-to-high grade steatosis group (mean difference [95% CI] 5.9 [2.2-9.6]%, Cohen’s d = 0.75), and (0.7 [0.3-1.1], Cohen’s d = 0.80, respectively). There was no association between LFF, as a categorical or continuous variable, and MinMod SI. The proportions of patients with none to mild fibrosis, moderate fibrosis and severe fibrosis were 14, 78 and 6%, respectively, and there were no significant associations between level of fibrosis and measures of insulin sensitivity.

**Conclusions:**

Patients with morbid obesity and T2DM demonstrated high levels of liver fat fraction, and we showed that hepatic steatosis, but not the degree of liver fibrosis, was associated with different measures of insulin sensitivity in patients with severe obesity and T2DM. Further, our results might indicate that the LFF is primarily associated with hepatic, and not peripheral insulin sensitivity. To improve the diagnosis of NAFLD and the prediction of its progression, more studies are needed to reveal the pathological mechanistic pathways involved in NAFLD and insulin sensitivity.

**Trial registration:**

ClinicalTrials.gov: NCT01778738

## Background

As the obesity epidemic soars, the prevalence of associated medical problems such as type 2 diabetes mellitus (T2DM), cardiovascular diseases, dyslipidemia and non-alcoholic fatty liver disease (NAFLD) increases steadily, and add to a solid rise of the health-care costs worldwide [1, 2]. NAFLD, which includes hepatic steatosis, steatohepatitis, fibrosis and cirrhosis, is the most frequent chronic liver disorder with a global prevalence of 25% [3]. Thus, NAFLD renders a serious health issue as it may progress into adverse conditions such as liver failure and hepatocellular carcinoma [2, 4].

The pathogenesis of NAFLD may be explained by the liver’s restricted capacity to handle large amounts of carbohydrates and fatty acids. Increased dietary intake of fat and carbohydrates may lead to increased levels of blood free fatty acids from the adipose tissue and increased de novo lipogenesis leading to toxic lipids caused by oxidative stress and inflammation. Hence, this could cause hepatocellular injury, fibrosis, cirrhosis and hepatocellular carcinoma [5]. Reduced insulin sensitivity, which is often observed in persons with NAFLD, may boost hepatic fat accumulation by increasing free fatty acid release from adipose tissue, and by the effect of hyperinsulinemia on anabolic processes [6]. However, the complete underlying mechanisms of the progression from hepatic steatosis to steatohepatitis and fibrosis are not fully understood [7, 8]. NAFLD has been shown to double the risk of incident T2DM [9, 10], and NAFLD is present in 60 to 90% of patients with obesity and T2DM [2]. Patients with T2DM and NAFLD have more severe hyperinsulinemia, dyslipidemia and lower insulin sensitivity in hepatic and adipose tissue, compared with patients without NAFLD [11]. Advanced liver fibrosis is also common in patients with T2DM, affecting at least one out of six patients with T2DM [12]. Altogether, obesity, T2DM, NAFLD and insulin resistance are pathological conditions highly linked to each other and possible to be considered part of a general metabolic dysregulation, but this relationship is not fully elucidated. Hence, we aimed to assess the association of hepatic steatosis and fibrosis with different measures of hepatic and peripheral insulin sensitivity in patients with severe obesity and T2DM.

## Methods

### Study design and participants

This is a cross-sectional analysis of baseline data from the Oseberg study, an ongoing single center, randomized controlled trial (NCT01778738) designed to compare the effects of Roux-en-Y gastric bypass and sleeve gastrectomy on remission of T2DM and β-cell function. The study was conducted at the Morbid Obesity Centre at Vestfold Hospital Trust, a tertiary care obesity center in Southern Norway between January 2013 and February 2018. A protocol article describing the study design and setting has been published [13]. The study was approved by the Regional Committees for Medical and Health Research Ethics in Norway (2012/1427/REK sør-øst B). In brief, patients with T2DM scheduled for bariatric surgery were screened for study eligibility according to the following criteria: age 18 years or older; current body mass index (BMI) of 33.0 kg/m^2^ or higher with previously verified BMI of 35.0 kg/m^2^ or higher; and T2DM (HbA1c of ≥6.5% [48 mmol/mol] or use of antidiabetic medications with HbA1c of ≥6.1% [43 mmol/mol]). The key exclusion criteria were previous major abdominal surgery, cancer, severe medical conditions associated with increased risk of complications, drug or alcohol addiction, pregnancy, and severe gastro-esophageal reflux disease. All patients were screened for hepatitis B and C through serological testing.

### Variables and data measurement

#### Hepatic steatosis

Hepatic steatosis was assessed by measuring the liver fat fraction (LFF) and by calculating the fatty liver index (FLI). To quantify the LFF we applied magnetic resonance imaging (MRI) (Siemens AERA 1.5 T) and chemical shift imaging [14], giving two groups of images in the same spatial domain with the water and fat spins in-phase or out-of-phase, thus being able to produce separate water and fat images. To quantify the fat content we used the modified Dixon method [15]. The fat signal percentage (FSP) in the liver was calculated as; FSP = [(SIT1 IP - SIT1 OP)/2(SIT1 IP)] * 100, where SIT1 IP is the ratio of hepatic signal intensity to splenic signal intensity on in-phase T1-weighted images, and SIT1 OP is the ratio of hepatic signal intensity to splenic signal intensity on out-of-phase T1-weighted images. The signal intensity in liver was normalized to spleen. A normal liver usually has a fat content of less than 5% histologically [16]. Based on reported studies, MRI has a sensitivity of 77-100% and a specificity of 87-91% for detection of any degree of hepatic steatosis (histologic grade > 5%) [17]. Imaging-based methods as the modified Dixon, allow detection of a fat percentage of 10-15 or more [18]. Two independent experienced consultant radiologists (NPK and JOG) performed the MRI analyses. They were unaware of all clinical and biochemical parameters of the study subjects. No or low- grade hepatic steatosis was defined as values below the 25^th^ percentile at baseline, which corresponded to a LFF ≤10%.

Fatty liver index (FLI) is an algorithm developed to predict fatty liver in the general population. Based on BMI, waist circumference (WC), triglycerides (TG) and gamma-glutamyl-transferase (GGT) this algorithm has an accuracy of 0.84 (95%CI 0.81- 0.87) in detecting fatty liver [19]. It was initially validated against ultrasonography. The score is calculated using the following formula:

FLI = (e^[0.953 × ln(TG) + 0.139 × BMI + 0.718 × ln(GGT) + 0.053 × WC - 15.745] / (1 + e^[0.953 × ln(TG) + 0.139 × BMI + 0.718 × ln(GGT) + 0.053 × WC - 15.745]) × 100, with TG measured in mg/dl (1 mg/dl = 0.01129 mmol /l), GGT in U/l, and WC in cm. The FLI score range is 0–100. Applying cutoffs proposed by Bedogni et al., a FLI < 30 rules out and a FLI ≥60 rules in fatty liver [19]. The algorithm has also been validated against proton magnetic resonance spectroscopy [20].

#### Liver fibrosis

To estimate the level of liver fibrosis, we used the enhanced liver fibrosis (ELF) test and calculated the NAFLD fibrosis score. The enhanced liver fibrosis (ELF) test is a proven and less invasive direct marker for evaluating liver fibrosis [21, 22]. Serum biomarkers of hepatic matrix metabolism including hyaluronic acid (HA), procollagen III amino terminal peptide (PIIINP) and tissue inhibitor of metalloproteinase 1 (TIMP1) are measured. A higher concentration of individual biomarkers leads to a higher ELF score and indicates a greater likelihood of more severe fibrosis. The ELF test has shown to correlate with the level of liver fibrosis assessed by liver biopsy [23]. The ELF score is calculated by the formula: ELF score = 2.278 + 0.851 × ln(HA) + 0.751 × ln(PIIINP) + 0.394 × ln(TIMP-1), all expressed in ng/ml [24]. A commercial kit from Siemens Healthineers was applied, ADVIA Centaur® XP Immunoassay System, and the analyses were performed at Unilabs Laboratoriemedisin, Oslo, Norway. The interpretation of the ELF score is as follows: < 7.7 (none to mild fibrosis), ≥7.7 to < 9.8 (moderate fibrosis) and ≥ 9.8 (severe fibrosis) [21, 23].

NAFLD fibrosis score was calculated using the following formula: − 1.675 + 0.037 × Age (yrs) + 0.094 × BMI (kg/m^2^) + 1.13× impaired fasting glucose/diabetes (yes = 1, no = 0) + 0.99 × Aspartate transaminase (AST)/ Alanine aminotransferase (ALT) ratio − 0.013 × Platelet(× 10^9^/L) − 0.66 × Albumin (g/dl). The interpretation of NAFLD fibrosis score is as follows: <− 1.455 = F0-F2 (no fibrosis to moderate fibrosis), − 1.455 – 0.675 = indeterminant score and > 0.675 = F3-F4 (severe fibrosis to cirrhosis) [25].

#### Insulin sensitivity

Insulin sensitivity was calculated in three different ways. First, the Homeostasis Model Assessment 2 (HOMA2S) calculate insulin sensitivity from fasting C-peptide and glucose levels using the HOMA2 calculator [26, 27]. HOMA2S estimates insulin sensitivity as percentage of a normal reference population. Second, the Matsuda insulin sensitivity index (Matsuda ISI) calculate insulin sensitivity from fasting and stimulated insulin and glucose levels obtained from the 3 h oral glucose tolerance test (OGTT) [28]. Finally, an insulin-modified intravenous glucose tolerance test (IGTT) was done as previously described [13]. Insulin sensitivity was derived using the MinMod Millennium Program version 6.02.16. For the calculations, a specific weighting algorithm was adopted for improving the fit of the model to the data [29].

#### Laboratory tests

Laboratory analyses were performed at the Central Laboratory, Vestfold Hospital Trust, which is accredited according to NS-EN ISO 15189 and serves as the main analytical facility in the hospital. General clinical chemistry and immunochemistry were analyzed on Cobas 8000 with modules ISE, c702 and e801 (Roche Diagnostics, Basel, Switzerland). Insulin were analyzed at Oslo University Hospital using established methods. A more detailed description of the laboratory analyses has previously been described [13]. Whole blood HbA1c was analyzed on a Tosoh high-performance liquid chromatography G8 analyser (Tosoh Corporation, Tokyo, Japan) with reagents from supplier. Hepatitis serology was analyzed on Cobas 8000 on module e801 with reagents from the supplier (Roche Diagnostics, Mannheim, Germany).

#### Study size

The sample size was calculated according to the primary outcomes [29], and the study sample was set to 125 participants given a 5% significance level and 80% power. Of the 125 patients enrolled at baseline examination, 109 were randomized with 100 of these patients undergoing MRI.

### Statistical analyses

Between-group comparisons of demographic and clinical characteristics were performed using independent-samples t-test, Mann-Whitney Wilcoxon test, ANOVA or Chi-square tests as appropriate. The results were reported as means (standard deviation [SD]), median (interquartile ranges [IQRs] or counts [percentages]). Estimation of effect sizes were performed by using Cohen’s d. Comparison of groups with different sample size was calculated by adjusting the pooled standard deviation with weights for the sample sizes. Cohen’s d values between 0.2 and 0.3 were considered to be a small effect size, medium effects were assumed for values around 0.5 and values larger than 0.8 would depict large effects. The statistical analyses were performed using SPSS Statistics 26 or STATA SE version 16.0.

## Results

### Participant flow and baseline characteristics

**Fig. 1:**
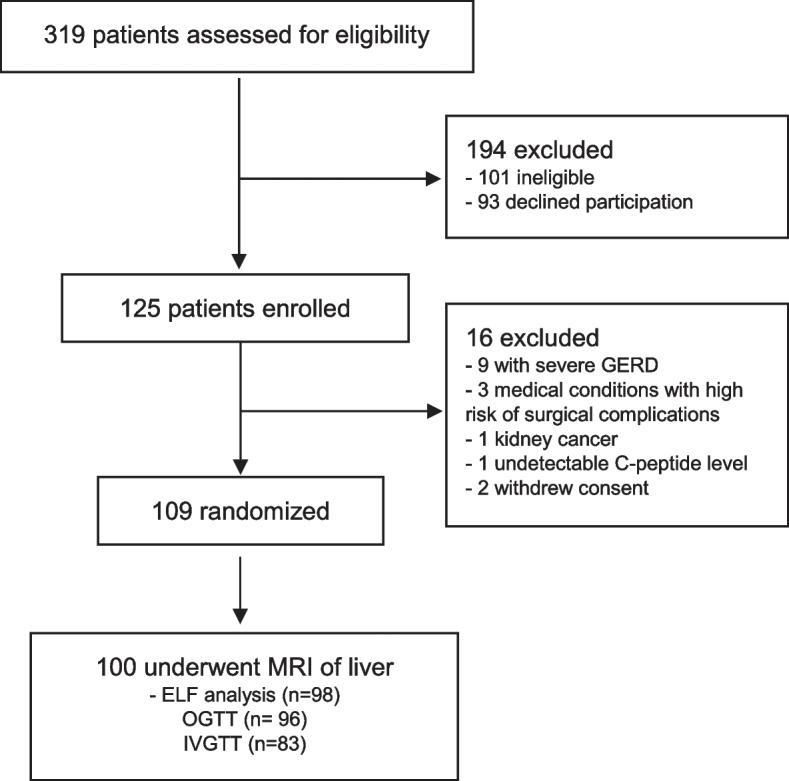
Schematic overview of selection of participants included in the current study

Between October 2012 and September 2017, 319 consecutive patients with severe obesity and T2DM were assessed for eligibility, of whom 194 were ineligible or declined participation. Thus, 125 patients were initially enrolled and underwent a baseline examination between January 2013 and February 2018. Of these patients, 16 were excluded mainly due to severe gastroesophageal reflux disease or medical conditions with high risk of surgical complications, leaving 109 patients scheduled for bariatric surgery in the Oseberg trial. Nine patients did not go through MRI due to claustrophobia or metal implants at baseline, leaving 100 patients to be included in the current cross-sectional analysis (Fig. 1). The results of the ELF test were available in 98 of the 100 patients. There were two fallouts due to analysis failure.

The participants (97% of White ethnicity, 65% female) had a mean (standard deviation [SD]) age of 47.5 (9.7) years, a BMI of 42.0 (5.3) kg/m^2^ and a WC of 128 (12) cm. Ninety-eight percent had metabolic syndrome with 89 and 65% using antidiabetic and antihypertensive medication, respectively. The mean (SD) alcohol intake was 1.0 (1.4) units a week. The mean LFF, as measured by MRI, was 19.1 (11.5) %, and the mean FLI-value was 53 (26), close to the cut-off ≥60 ruling in fatty liver. The mean (SD) ELF score was 8.46 (0.84), a value indicating moderate fibrosis.

### Measures of insulin sensitivity and clinical characteristics by level of hepatic steatosis

**Fig. 2:**
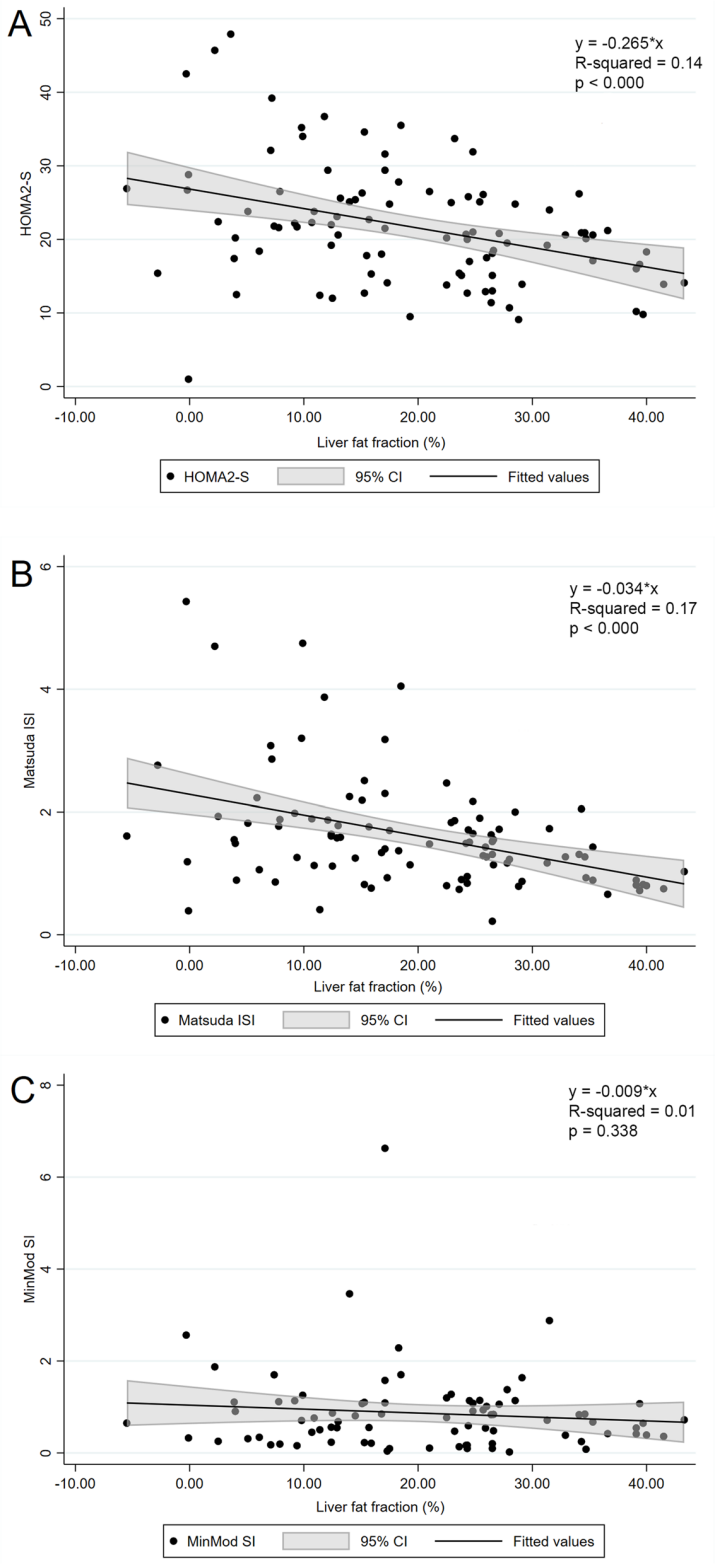
Scatterplots with fitted linear regression line with 95% CI, showing the association between measures of insulin sensitivity and levels of liver fat fraction (%) estimated by MRI. **A** HOMA2S, **B** Matsuda ISI and **C** MinMod SI

The mean LFF was 5 (4) % and 24 (9) % in the no or low-grade steatosis group and medium-to-high grade steatosis group, respectively (Table 1). The mean fasting C-peptide was significantly lower in the no or low-grade steatosis group compared with the medium-to-high grade steatosis group (mean difference [95% confidence interval (CI)]: − 422 [− 641 to − 204] pmol/L, *p* < 0.001, *[*Cohen’s d] = 0.92). HOMA2S and Matsuda ISI were significantly higher in the no or low-grade steatosis group compared with the medium-to-high grade steatosis group (mean difference [95% CI] 5.9 [2.2 to 9.6] %, Cohen’s d = 0.75), and (0.7 [0.3 to 1.1], Cohen’s d = 0.80, respectively). LFF was inversely associated with HOMA2S (Fig. 2A) and Matsuda ISI (Fig. 2B), and for every 1% increase in LFF, the HOMA2S was reduced with 0.5% and Matsuda ISI reduced with 0.03. There was no association between LFF, as a categorical or continuous variable, and MinMod SI (Table 1, Fig. 2C).

**Table 1:** Clinical characteristics according to grade of steatosis based on liver fat fraction measured by MRI

	All	No or low grade steatosis^**a**^	Medium to high grade^**a**^	***P***-value
N (%)	100 (100)	25 (25)	75 (75)	
Liver fat fraction, % (range)	19.1 (−5.5–43.3)	4.7 (− 5.5–9.9)	24.2 (10.7–43.3)	< 0.001
Age, years	47.5 (9.7)	49.3 (8.8)	46.9 (10.0)	0.297
Gender, female (%)	65	19 (76)	46 (61)	0.183
Metabolic syndrome, n (%)	98 (98)	25 (100)	73 (97)	0.409
White ethnicity, n (%)	97 (97)	24 (96)	73 (97)	0.735
Alcohol consumption, units a week	1.0 (1.4)	0.7 (1.0)	1.1 (1.5)	0.229
***Antropometrics***				
BMI, kg/m^2^	42.0 (5.3)	41.6 (5.8)	42.1 (5.1)	0.688
FFM, kg	66.7 (13.9)	64.5 (11.9)	67.4 (14.5)	0.364
FM, kg	58.7 (12.7)	58.4 (13.6)	58.8 (12.5)	0.886
Waist circumference, cm	127.8 (11.9)	126.3 (13.4)	128.2 (11.4)	0.489
***Glucose homeostasis***				
Fasting glucose, mmol/L	12.1 (4.6)	12.8 (5.2)	11.8 (4.4)	0.329
HbA1c, mmol/mol	66.2 (18.7)	65.5 (13.0)	66.5 (20.4)	0.831
Fasting insulin, pmol/L	199 (133)	159 (129)	212 (132)	0.079
Fasting C-peptide, pmol/L	1600 (493)	1277 (554)	1700 (430)	< 0.001
HOMA2S, %	21.8 (8.2)	26.3 (11.0)	20.4 (6.6)	0.002
Matsuda ISI	1.6 (0.9)	2.2 (1.3)	1.5 (0.7)	< 0.001
MinMod SI, (mu/l)^−1^ x min^− 1^	0.86 (0.90)	0.87 (0.69)	0.86 (0.95)	0.981
Diabetes duration, years	6.7 (6.1)	10.4 (7.5)	5.5 (5.1)	< 0.001
Antidiabetic medication, n (%)	89 (89)	23 (92)	66 (88)	0.580
***Liver status***				
Alanine aminotransferase (U/L)	38.5 (20.6)	26.2 (13.0)	42.6 (21.1)	< 0.001
Aspartate aminotransferase (U/L)	27.7 (12.5)	21.8 (8.8)	29.7 (12.9)	0.006
AST/ALT ratio	0.79 (0.26)	0.89 (0.27)	0.75 (0.24)	0.027
ƴ-Glutamyltransferase (U/L)	56.1 (37.8)	43.1 (28.7)	60.4 (39.6)	0.048
ELF-score	8.46 (0.84)	8.37 (1.04)	8.49 (0.77)	0.551
Fatty Liver Index	52.6 (25.8)	43.5 (27.1)	55.6 (24.8)	0.041
NAFLD Fibrosis score	−0.025 (0.922)	0.082 (0.966	−0.061 (0.911)	0.505
***Cardiovascular risk factors***				
Total cholesterol, mmol/L	4.4 (0.9)	4.3 (0.7)	4.5 (0.9)	0.421
LDL cholesterol, mmol/L	2.5 (0.8)	2.4 (0.6)	2.5 (0.8)	0.482
HDL cholesterol, mmol/L	1.0 (0.2)	1.1 (0.2)	1.0 (0.2)	0.007
Triglycerides, mmol/L	2.1 (1.3)	1.7 (0.7)	2.3 (1.4)	0.065
Systolic BP, mmHg	132.3 (14.9)	136.8 (19.8)	130.7 (12.6)	0.078
Diastolic BP, mmHg	84.5 (7.1)	84.9 (7.0)	84.4 (7.2)	0.766
Antihypertensives, n (%)	65 (65)	20 (80)	45 (60)	0.069
Lipid-lowering drugs, n (%)	46 (46)	15 (60)	31 (41)	0.105

Data are presented as n (%) and mean (SD). Liver fat fraction (LFF) was estimated with the modified Dixon method calculating the percentage of liver fat using magnetic resonance imaging (MRI). No or low grade hepatic steatosis was defined in patients with values below or equal to the 25th percentile (≤10%) of LFF at baseline. Two-sample t-test with equal variance for continues variables and Pearson Chi-squared for categorical variables. MinMod SI, *n* = 83; ELF, *n* = 98; Matsuda ISI, *n* = 96; C-peptide, *n* = 98

*FFM* Fat free mass, *FM* Fat mass

^a^No or low grade hepatic steatosis was defined in patients with values below or equal to the 25th percentile (≤ 10%) of LFF at baseline, while medium to high grade steatosis was defined as > 10%

The duration of diabetes was longer in the no or low-grade steatosis group compared with the medium-to-high grade steatosis group (10.4 (7.5) versus 5.5 (5.1) years, *p* < 0.001). Other clinical characteristics did not differ significantly between the groups (Table 1).

We observed significantly lower mean (SD) values of AST and ALT in the no or low-grade steatosis group compared with the medium-to-high grade steatosis group (Table 1). Comparing the no or low-grade steatosis group with the medium-to-high grade steatosis group (Table 1), there were no difference in degree of fibrosis using the ELF test or the NAFLD fibrosis score.

High density lipoprotein levels were higher (1.1 [0.2] versus 1.0 [0.2] mmol/L, *p* = 0.007) and the TG levels tended to be lower (1.7 [0.7] versus 2.3 [1.4] mmol/L, *p* = 0.065) in the no or low-grade steatosis group compared with the medium-to-high grade steatosis group (Table 1).

### Measures of insulin sensitivity and clinical characteristics by level of liver fibrosis

**Fig. 3:**
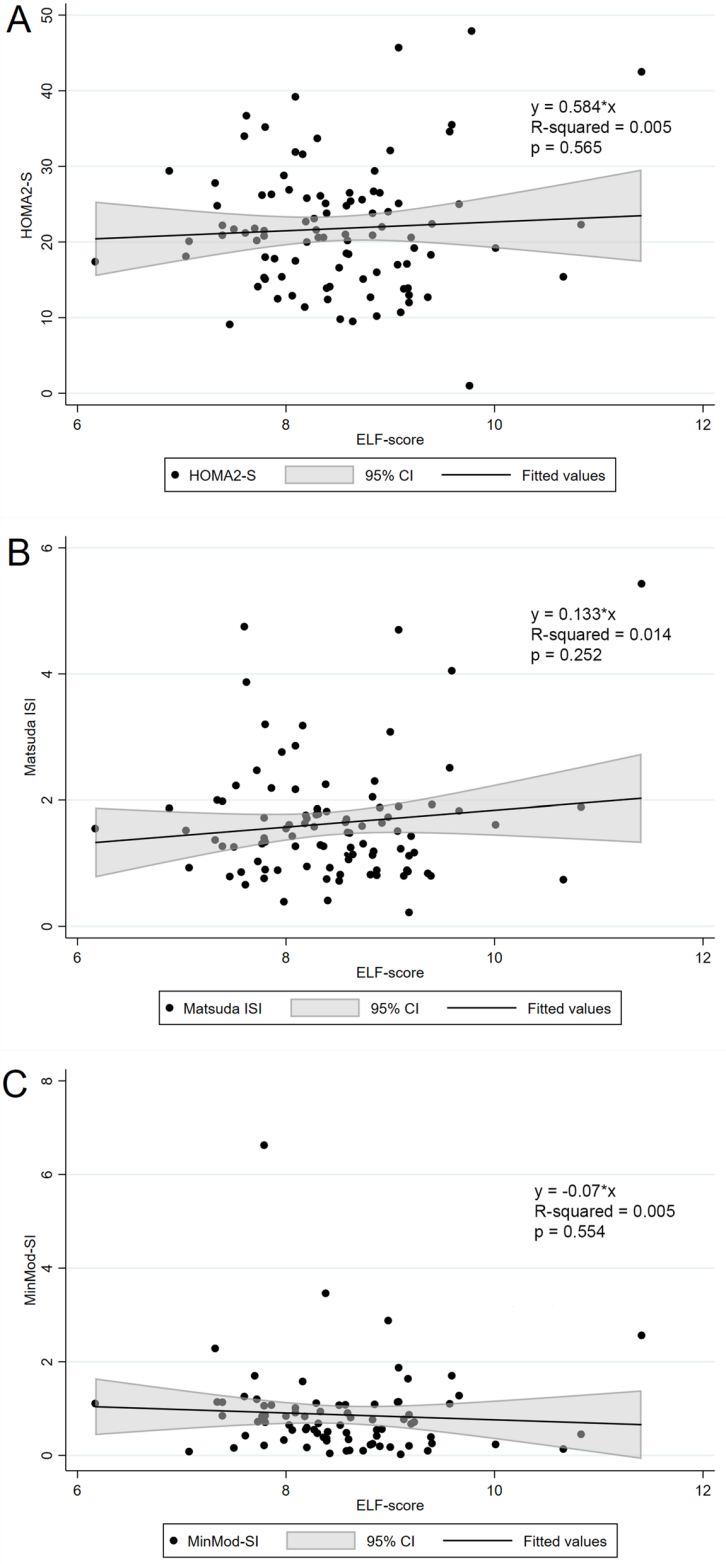
Scatterplots with fitted linear regression line with 95% CI, showing the association between measures of insulin sensitivity and levels of fibrosis assessed by ELF-score. **A** HOMA2S, **B** Matsuda ISI and **C** MinMod SI

The proportions of patients with none to mild fibrosis, moderate fibrosis and severe fibrosis were 14, 78 and 6%, respectively (Table 2). Insulin sensitivity measured by HOMA2S, Matsuda ISI and MinMod SI did not differ between the groups. There were no significant associations between the ELF-score and the different measures of insulin sensitivity (Fig. 3, panel A-C).

**Table 2:** Clinical characteristics according to level of fibrosis based on the Enhanced Liver Fibrosis (ELF™) test

	None to Mild	Moderate	Severe	***P***-value
	*ELF-score < 7.7*	*ELF-score* ≥ *7.7 to* ≤ *9.8*	*ELF score > 9.8*	
N (%)	14 (14)	78 (80)	6 (6)	
ELF-score	7.28 (0.39)	8.52 (0.54)	10.41 (0.67)	na
Age, years	39.8 (8.3)	48.4 (9.2)	54.3 (12.0)	0.002
Gender, female (%)	14 (100%)	46 (59%)	4 (67%)	0.012
Metabolic syndrome, n (%)	14 (100%)	76 (97%)	6 (100%)	0.77
White ethnicity, n (%)	14 (100%)	75 (96%)	6 (100%)	0.672
Alcohol consumption, units a week	0.3 (0.8)	1.1 (1.5)	0.9 (0.9)	0.155
***Antropometrics***				
BMI, kg/m^2^	43.3 (5.3)	41.3 (4.5)	44.5 (9.7)	0.15
FFM, kg	58.9 (6.4)	67.7 (14.4)	67.7 (17.0)	0.088
FM, kg	61.1 (11.6)	57.6 (11.4)	62.5 (22.4)	0.432
Waist circumference, cm	122.7 (11.8)	127.8 (10.6)	133.7 (18.8)	0.122
***Glucose homeostasis***				
HbA1c, mmol/mol	70.6 (18.8)	65.5 (19.3)	64.0 (15.4)	0.627
Fasting insulin, pmol/L	160 (92)	206 (143)	136 (75)	0.276
Fasting C-peptide, pmol/L	1313 (472)	1658 (475)	1370 (655)	0.04
HOMA2S, %	24.7 (12.9)	23.4 (11.2)	30.6 (16.7)	0.478
Matsuda ISI	1.83 (1.17)	1.55 (0.79)	2.42 (2.07)	0.134
MinMod SI, (mu/l)^−1^ x min^−1^	1.03 (0.65)	0.85 (0.93)	0.85 (1.15)	0.864
Diabetes duration, years	5.6 (7.2)	6.5 (5.5)	13.7 (8.3)	0.016
Antidiabetic duration, n (%)	12 (86)	69 (88)	6 (100)	0.638
***Liver status***				
ALAT, U/L	30.6 (19.7)	39.3 (20.1)	43.2 (29.1)	0.297
ASAT, U/L	22.9 (11.7)	27.4 (10.4)	41.3 (27.5)	0.009
AST/ALT ratio	0.80 (0.25)	0.78 (0.25)	0.97 (0.37)	0.168
GammaGT,	52.4 (42.4)	55.5 (36.1)	68.2 (54.2)	0.691
Liver fat fraction, %	19.2 (11.9)	19.7 (11.4)	8.3 (9.2)	0.066
Fatty Liver Index	49.2 (28.6)	51.5 (24.6)	61.2 (29.2)	0.617
NAFLD Fibrosis score	−0.65 (1.2)	0.02 (0.84)	0.79 (0.39)	0.003
***Cardiovascular risk factors***				
Total cholesterol, mmol/L	4.9 (1.3)	4.4 (0.8)	4.5 (0.5)	0.084
LDL cholesterol, mmol/L	2.9 (0.9)	2.5 (0.7)	2.5 (0.7)	0.163
HDL cholesterol, mmol/L	1.1 (0.2)	1.0 (0.2)	1.2 (0.2)	0.048
Triglycerides, mmol/L	2.2 (1.2)	2.0 (1.0)	2.0 (0.7)	0.856
Systolic BP, mmHg	129 (11)	132 (14)	147 (27)	0.034
Diastolic BP, mmHg	85 (7)	85 (7)	85 (8)	0.952
Antihypertensive medications, n (%)	8 (57)	50 (64)	5 (83)	0.532
Lipid-lowering drugs; n (%)	5 (36)	36 (46)	4 (67)	0.443

Data are presented as n (%) and mean (SD). Between group difference were performed using analysis of variance (ANOVA). MinMod SI, *n* = 83; ELF, *n* = 98; Matsuda ISI, *n* = 96; C-peptide, *n* = 98

*FFM* Fat free mass, *FM* Fat mass

The mean (SD) age for the patients with none to mild fibrosis, moderate fibrosis and severe fibrosis were 39.8 (8.3), 48.4 (9.2) and 54.3 (12.0) years, respectively (*p* = 0.002). Patients with severe fibrosis had a longer duration of T2DM (13.7 [8.3] years) compared with those with moderate fibrosis (6.5 [5.5]) and none or mild fibrosis (5.6 [7.2], *p* = 0.016). Patients with severe fibrosis also presented lower LFF (8 [9] %, *p* = 0.066) compared with none to mild fibrosis and moderate fibrosis (19 [12] and 20 [11] respectively *p* = 0.066, Table 2).

The mean (SD) NAFLD fibrosis score for patients with none to mild fibrosis, moderate fibrosis and severe fibrosis were − 0.65 (1.2), 0.02 (0.84) and 0.79 (0.39), respectively (*p* = 0.003), all values corresponding to the categories of moderate to severe fibrosis. Patients with none to mild fibrosis had lower mean (SD) values of AST 22.9 (11.7) compared with patients with moderate (27.4 (10.4) and severe fibrosis (41.3 (27.5), (*p* = 0.009), but there were no significant difference between the fibrosis-groups with regards to ALT values or AST/ALT ratio (Table 2).

## Discussion

The clinical and pathophysiological connections between NAFLD, insulin resistance, obesity and T2DM have been widely explored [30]. However, few studies, if any, have applied three different measures of insulin sensitivity in combination with MRI and the ELF-test for estimation of liver fat fraction and liver fibrosis, respectively, to assess the relationship between levels of hepatic steatosis and fibrosis with insulin sensitivity in patients with severe obesity and T2DM. Our data show that most (> 75%) of the patients with severe obesity and T2DM had moderate-to-high grade of liver steatosis, and there was a high proportion (80%) of patients with moderate fibrosis. With increasing LFF, we observed reduced insulin sensitivity measured by HOMA2S and Matsuda ISI. On the contrary, there was no association between LFF and intravenous glucose tolerance test-derived insulin sensitivity. Notably, the grade of fibrosis was independent of LFF, and we observed no associations between grade of fibrosis and the different measures of insulin sensitivity.

Similar to our study, a vast number of papers have reported a high prevalence of NAFLD in patients with both T2DM and obesity [1, 31]. The degree of hepatic steatosis is tightly linked to obesity and patients with obesity are simultaneously at increased risk of associated medical problems such as T2DM, dyslipidemia and hypertension, a cluster of metabolic conditions influencing the susceptibility for development of NAFLD. In a Finnish study of 140 patients, of which half of the patients had T2DM, patients with T2DM had 80% more liver fat compared with those without T2DM, when matched for age, sex and weight [32]. Several meta-analyses have also shown that NAFLD increases the risk the of incident T2DM [9, 33], and there is some evidence suggesting that patients with T2DM are at higher risk of developing liver fibrosis [31, 34, 35]. Thus, the high proportion of patients with steatosis and moderate to severe fibrosis in our cohort of patients with severe obesity and T2DM was a predictable finding.

Unfavourable fat distribution, adipose tissue dysfunctionality and insulin resistance constitute the basis of metabolic disturbances such as NAFLD [30]. Current literature suggests that the pathogenic drivers are not identical among all patients and includes multiple metabolic, genetic and microbiome related factors [22], thus the pathophysiological mechanisms behind the development of NAFLD seems to be best explained by the “multi-hit hypothesis” [36]. Insulin resistance within the liver and in the extra-hepatic tissue is implicated in the pathogenesis, and recent data indicate that hyperinsulinemia could be seen as both a consequence and a cause of NAFLD [37]. Hyperinsulinemia may cause increased de novo lipogenesis in the liver, and results in accumulation of ectopic fat in peripheral tissues, generating macrophage infiltration and a pro-inflammatory state that promotes insulin resistance [5, 22, 30, 38]. In line with this notion, HOMA2S which reflects the balance between hepatic glucose output and insulin secretion in the basal state and is a measure of mostly hepatic insulin sensitivity [39], was moderately associated with LFF. Similarly, Matsuda ISI which also includes fasting measurements, correlated well with LFF. In contrast, MinMod SI, which includes measurements 20 min after rise in glucose levels and reflects mostly glucose disposal in the skeletal muscles, did not correlate with LFF. Due to the cross-sectional design of this study, no casuality between liver fat content and insulin sensitivity can be established. However, our findings indicate that LFF is primarily associated with hepatic, and not peripheral insulin sensitivity. The clear differences in insulin sensitivity, observed in our study, between patients with medium-to-high grade steatosis and patients with no or low-grade steatosis, are comparable with a study by Lomonaco and colleagues [11]. Among 154 patients with obesity with or without T2DM, the suppression of free fatty acids during a euglycemic-hyperinsulinemic clamp, a measure of hepatic insulin sensitivity, was negatively correlated with intrahepatic TG content.

An interesting finding in our study was the significant difference in duration of T2DM between the groups of steatosis, with a shorter duration in the medium-to-high grade steatosis group compared with the no or low-grade group. In line with this observation, a shorter duration of T2DM was found to be an independent predictor of NAFLD in a large cohort study from Scotland [40]. The association of shorter duration of T2DM and NAFLD is assumed to be caused by a greater degree of hyperinsulinemia in early TD2M as the hyperinsulinemia may drive the uptake of free fatty acids in the hepatocytes [41]. In addition, long duration of T2DM may also reflect earlier diabetes debut or high age, which both indicate impaired beta cell function. It is therefore possible that patients with no or low-grade steatosis have a higher degree of hepatic insulin sensitivity, but a greater degree of beta cell dysfunction.

In patients with both severe obesity and T2DM, the co-existence of these conditions increases the risk of liver fibrosis due to the imbalance in the lipid metabolism and the formation of lipotoxic lipids, cellular stress, inflammation and cell death. Published data among patients with T2DM suggest a prevalence of biopsy proven advanced fibrosis globally of 17% [31]. With non-invasive methods the prevalence is estimated to be up to 37% [34]. Several studies have described association between T2DM and liver fibrosis, and to some extent, predictive factors associated with the development of fibrosis [34]. A study by Petta et al. reported that the risk of severe fibrosis, discriminated by age, was driven by low HDL, impaired fasting glycemia/diabetes and obesity at lower age, while impaired fasting glycemia/diabetes and low HDL were predictors of severe fibrosis at older age [42]. The major part of patients in our study were reported to have moderate fibrosis and there were no difference between the groups of fibrosis regarding HDL levels. We observed that patients with severe fibrosis were older, had longer duration of T2DM and had higher levels of AST. Both AST and ALT showed linkage to liver fat content, presenting higher values in the medium to high-grade steatosis group, but only AST demonstrated a significant difference between the different groups of fibrosis. Patients with advanced fibrosis may have normal range of liver enzymes [43]. Our findings supports the study by Mansour et al. [34] showing that AST, but not ALT and the AST/ALT ratio, was positively associated with fibrosis. However, it is important to note that normal values of liver function enzymes do not rule out the possibility of a significant stage of fibrosis [43, 44].

In addition to the ELF test, which is a proprietary fibrosis panel based on extracellular matrix proteins, we also applied the non-invasive NAFLD fibrosis score for assessment of fibrosis, a score developed specifically for NAFLD considering the parameters age, hyperglycemia, BMI, platelet count, albumin, and AST/ALT ratio. The ELF test has shown to be a better tool for assessment of fibrosis stage in NAFLD compared with the NAFLD fibrosis score [45], but the NAFLD fibrosis score largely matched the results of the ELF test in our study.

Our study had some limitations. Most patients were of White ethnicity (97%), thus the results may not be generalizable to other ethnicities. There is a moderate number of participants in the study. MRI with the modified Dixon method for estimation of LFF have demonstrated reduced accuracy when there is a low degree of steatosis in the liver. LFF values may even be negative as the signal intensity in liver is normalized to spleen. Hence, our study would have benefited from liver biopsy proven validation of the LFF values, especially for those in the no or low-grade steatosis group. Nevertheless, compared to a liver biopsy, MRI is non-invasive, without risk of pain, bleeding or risk of infection. The strength in our findings regarding hepatic steatosis and insulin sensitivity is the use of several insulin sensitivity indices, recognizing that HOMA2S primarily is an insulin sensitivity-surrogate evaluating NAFLD for patients without T2D [46]. The European Liver Fibrosis study group has validated the ELF score. By now it is an expensive test, but it has shown good performance in differentiating fibrosis stages in NAFLD, with a better accuracy when combined with the NAFLD fibrosis score (over 90% in distinguishing severe fibrosis) [47]. However, the performance can be influenced by age and gender [48].

## Conclusion

Our results showed that hepatic steatosis, but not the degree of liver fibrosis, was associated with different measures of insulin sensitivity in patients with morbid obesity and T2DM. The observed discrepancy between IGTT derived insulin sensitivity and measures of insulin sensitivity based on OGTT might indicate that the LFF is primarily associated with hepatic, and not peripheral insulin sensitivity. Patients with short duration of T2DM tended to have more steatosis while those with longer duration were more frequently presented with higher levels of fibrosis. To improve the diagnosis of NAFLD and the prediction of its progression, more studies are needed to reveal the pathological mechanistic pathways involved in NAFLD and insulin sensitivity in patients with obesity and T2DM.

## Data Availability

Access to data collected from this study, including anonymized individual-participant data, will be made available following publication upon e-mail request to the corresponding author (Kathrine Aglen Seeberg, UXSEEK@siv.no). After approval of a proposal, data will be shared with investigators whose proposed use of the data has been approved by the Oseberg steering committee, according to the consent given by the participants and Norwegian laws and legislations.

## Abbreviations

ALT: Alanine aminotransferase
AST: Aspartate aminotransferase
BMI: Body mass index
CI: Confidence interval
ELF: Enhanced liver fibrosis
FLI: Fatty liver index
GGT: Gamma-glutamyl-transferase
HDL: High density lipoprotein
HOMA2S: Homeostasis Model Assessment 2
IGTT: Intravenous glucose tolerance test
LFF: Liver fat fraction
MRI: Magnetic resonance imaging
NAFLD: Non-alcoholic fatty liver disease
OGTT: Oral glucose tolerance test
OSEBERG: Obesity *S*urg*e*ry in Tønsberg
SD: Standard deviation
T2DM: Type 2 diabetes mellitus
TG: Triglycerides
WC: Waist circumference
